# Comparison of household socioeconomic status classification methods and effects on risk estimation: lessons from a natural experimental study, Kisumu, Western Kenya

**DOI:** 10.1186/s12939-022-01652-1

**Published:** 2022-04-09

**Authors:** Vincent Were, Louise Foley, Eleanor Turner-Moss, Ebele Mogo, Pamela Wadende, Rosemary Musuva, Charles Obonyo

**Affiliations:** 1 Center for Global Health Research, Kenya Medical Research Institute, P O Box 1578-40100, Kisumu, Kenya; 2 MRC Epidemiology Unit, School of Clinical Medicine, University of Cambridge, Cambridge, UK; 3 Kisii University, Kericho, Kenya

**Keywords:** Inequalities inequity socioeconomic status classification methods hypermarket

## Abstract

**Introduction:**

Low household socioeconomic status is associated with unhealthy behaviours including poor diet and adverse health outcomes. Different methods leading to variations in SES classification has the potential to generate spurious research findings or misinform policy. In low and middle-income countries, there are additional complexities in defining household SES, a need for fieldwork to be conducted efficiently, and a dearth of information on how classification could impact estimation of disease risk.

**Methods:**

Using cross-sectional data from 200 households in Kisumu County, Western Kenya, we compared three approaches of classifying households into low, middle, or high SES: fieldworkers (FWs), Community Health Volunteers (CHVs), and a Multiple Correspondence Analysis econometric model (MCA). We estimated the sensitivity, specificity, inter-rater reliability and misclassification of the three methods using MCA as a comparator. We applied an unadjusted generalized linear model to determine prevalence ratios to assess the association of household SES status with a self-reported diagnosis of diabetes or hypertension for one household member.

**Results:**

Compared with MCA, FWs successfully classified 21.7% (95%CI = 14.4%-31.4%) of low SES households, 32.8% (95%CI = 23.2–44.3) of middle SES households, and no high SES households. CHVs successfully classified 22.5% (95%CI = 14.5%-33.1%) of low SES households, 32.8% (95%CI = 23.2%-44.3%) of middle SES households, and no high SES households. The level of agreement in SES classification was similar between FWs and CHVs but poor compared to MCA, particularly for high SES. None of the three methods differed in estimating the risk of hypertension or diabetes.

**Conclusions:**

FW and CHV assessments are community-driven methods for SES classification. Compared to MCA, these approaches appeared biased towards low or middle SES households and not sensitive to high household SES. The three methods did not differ in risk estimation for diabetes and hypertension. A mix of approaches and further evaluation to refine SES classification methodology is recommended.

**Supplementary Information:**

The online version contains supplementary material available at 10.1186/s12939-022-01652-1.

## Introduction

Socioeconomic status (SES) is associated with health-related behaviours, including diet. However, many studies that examine the role of SES and health disparities have provided inconsistent results partly due to complexity in methodology of measuring SES [1]. A recent systematic review of SES and risk factors for non-communicable disease in low- and lower-middle-income countries found that low SES groups consumed less fruit, vegetables, fish, and fibre than high SES groups, but contrastingly that high SES groups consumed more fats, salt, and processed food [2]. Similarly, in these settings SES is associated with risk of non-communicable disease, including greater risk of cancer and cardiovascular disease in low SES groups, but greater risk of diabetes in high SES groups [3]. SES is also a key factor influencing access to and utilization of health services and can produce health inequalities [4]. Households and individuals of lower SES are more likely to suffer from poorer health because of fewer resources [5]. However, these households may employ a variety of other coping strategies such as receiving help from family or friends, free social services, and selling their assets.

One way that household SES can influence diet is through purchasing practices, such as supermarkets or other food retail [6]. Proximity to supermarkets has a significant influence on the diet of the surrounding population [7]. Some studies have also shown that living closer to a supermarket also protects children's dietary behaviour and body mass index(BMI) [8–10]. This helps families with budget and cost constraints to obtain healthy food and modification of BMI by accessing affordable healthy food and beverages from these establishments. While higher SES has been associated with the risk of obesity and hypertension, economic constraints especially for those in low SES also prevents households from accessing cheaper healthy foods [11].

Because of the association between SES with health behaviours and outcomes, there is a need to assess methods of classification of households into SES strata (i.e. low, middle and high). This is particularly important in low- and middle-income countries (LMICs) currently experiencing a burgeoning burden of disease related to non-communicable disease and its relationship to the nutritional transition [12]. However, there is limited experience of stratification of SES in LMICs whereby the strengths and weaknesses of different classification systems are likely to vary across contexts [13]. Current measures have been shown to have poor inter-rater and test–retest reliability [14]. A recent study established that in a case–control study, if exposure and disease or both are misclassified, the association measures such as odds ratio (OR) and conclusions will be biased [15] and may lead to invalid results [16]. Econometric methods have been adopted in the classification of household SES such as principal component analysis (PCA) or multiple correspondent analysis (MCA) and these remain options, especially in resource-limited settings where other available qualitative approaches methods may be costly for the study [17, 18]. Other qualitative or community-based strategies have been adopted to identify poor households with varying degrees of bias [19, 20]. Limited studies have compared the accuracy and sensitivity of classification of households into SES compared two community-based approaches of using community health volunteers and trained research assistants and an econometric model largely driven by statistical methods of SES classification.

In this analysis, we, therefore, aimed to compare three methods of assessing household SES using data obtained from a natural experimental study [21] in an urban African city – Kisumu, in Western Kenya – with the end goal of understanding the sensitivity, specificity, inter-rater reliabilities, proportion of SES misclassifications and accuracy of three methods of household SES classification and the effect associated with the risk estimation of self-reported diabetes and hypertension.

## Materials and methods

### Study site and population

The study was conducted in Kisumu County of Western Kenya. Kisumu County had 968,909 individuals during the 2009 census [22]. The total coverage area is 2085.9km^2^ and 1113 M altitude above sea level. This analysis is nested in a natural experimental study assessing the potential effects of a new hypermarket (supermarket combined with department store) on diet-related outcomes in residents. The study was conducted within a 2 km radius of the Kisumu Lake Basin Mall (which houses the hypermarket) [21]. The mall, funded through a public–private partnership and currently planned to be the largest in Western Kenya, is located approximately 5 km from the central business district of the city of Kisumu, the seat of Kisumu County. The mall is planned to house other amenities, such as an Amphitheatre, doctors’ offices, cafeterias, a gym, and office spaces. Lake Basin Mall is not only in close proximity to a higher-SES residential area but also close to lower-SES informal (ie, slum) settlements. It is situated along a busy highway that opens up the western part of Kenya, runs past Kisumu International Airport, and continues toward Uganda's Northern Transport Corridor, a popular destination for Kenyan traders in commodities such as fish, fruit, cereals [21]

The 2 km radius was approximated to be the region households are likely to access the hypermarket for food purchases. Another radius of 1 km and 0.5 m was also drawn around the mall to allow verifying distance to the mall. The research area was mapped and updated into a geolocation information system in the CommCare mobile application [23]. The study area was also divided into four quadrants: north-east, north-west, south-east, and south-west to enable ease of data collection.

### Sampling procedure

We worked with the department of health in Kisumu County which has developed a functional community health unit (CHU) through their community health strategy [24]. Through the system, community health volunteers (CHVs) are the first level of care and link households to health facilities. In a functional CHU, CHVs are trained and hold regular meetings with representatives of the villages, are provided with supportive supervision by the community health extension workers (CHEWS). In a functional CHU, there exists a community-based health information system (CBHIS) for capturing data. regular health dialogue days are helpful and the community members often agree to action days to implement suggestions from the dialogue meetings [25]

A total of 20 CHVs working within a 2 km radius of the Lake Basin Mall helped us to generate lists of about 2000 households in the study area [21]. We then used a stratified sampling technique (probability proportionate to size) to randomly sample by household SES (low, middle and high – classification described in more detail below), distance (within 0.5 km, 1 km and 2 km from the mall) and quadrant (NE, NW, SE and SW). The final sample comprised 200 households estimated from the main protocol which assumed a 5% household food expenditure share, 80% power, 95% confidence interval and a 30% attrition rate.

**Fig. 1:**
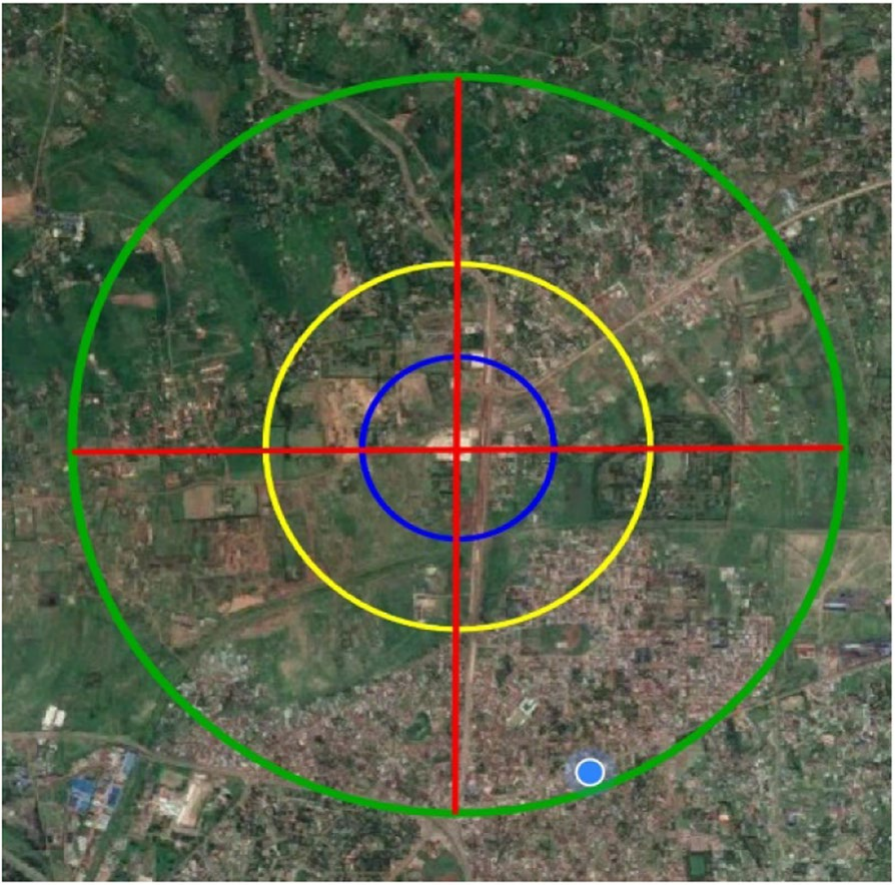
Map showing the study area is shown below with a radius of 2 km ( green line), 1.5 m ( yellow line) and 0.5 km (blue line) around the Lake basin Mall, the study landmark

Figure 1 shows the distribution of households and the regions of sampling around the Kisumu Lake basin Hypermarket.

### Methods of SES classification

SES classification literature and different classification tools for asset-based SES were reviewed. This was followed by a site visit to different study areas by researchers, further discussion in stakeholder workshops to select and develop a pragmatic asset-based classification tool. Training on SES classification with field workers (FWs) and CHVs was based on the indicators described in Table 1 [13]. The FWs attended the research training which lasted five days while CHVs had a one-day meeting to discuss SES classifications. These classifications were broadly based on asset-based measures used in previous household surveys conducted in LMICs [13]. FWs were offered photos taken by lead researchers during site visits in November 2019 as examples of households of different SES and asked to classify them. Study teams then had this classification with them when visiting households to ensure consistency in data collection.

**Table 1:** Examples given to study teams of asset indicators for low, middle and high socioeconomic households based on characteristics of dwelling [26, 27]

Socioeconomic status	Examples based on local knowledge of the study areas
Low	• Roof—grass thatch, *makuti*, tin cans • Walls—mud, canvas • Floor—earth, mud, dung, sand • Source of water—well, dam/ untapped water source • Lack of electricity • Sanitation—no latrine, shared facility, pit latrine • Condition of the house • Construction materials for the door and the window • Household gadgets • Average per capita income per month • Possessed land/household profile
Middle	• Roof—corrugated iron (*mabati*) • Walls—bricks • Floor—concrete, tiles • Source of water—tap outside/inside the house • Presence of electricity • Sanitation—VIP, flush toilet • Housing condition • Door and window construction materials • Household gadgets • Average per capita per month • Possessed land/household profile
High	• Roof—asbestos sheets, concrete, tiles • Walls—stone, concrete blocks • Floor—tiles, polished wood • Source of water—tap inside the house • Presence of electricity • Sanitation—flush toilet • Housing condition (permanent house) • Construction materials for the door and the windows • Average per capita per month • Possessed land/household profile

### Indicators of socioeconomic status of households

Table 1 shows indicators of SES which describes elements which were used by CHVs to classify households into low, middle, and high SES and a sample frame of 2000 households.

#### Community health volunteers

In the Kenyan health system, CHVs represent the first basic level of care. They are volunteers with the majority having completed primary education (8 years of education), and are elected or nominated by their communities to serve them on health-related matters under the community health strategy [25]. A CHV typically works in a village with about 100 households and is supervised by community health assistants [28], who are government employees [29]. CHVs visit households monthly to collect data on general health indicators and often work with partners in their respective areas. They are assumed to know the households well.

To assist the research team to undertake sampling, each of the 20 CHVs provided a list of about 100 households from their villages, up to a total of about 2000 households. Typically, each CHV visits 100 households monthly and have a register that has all the names of household heads. We provided the CHV with a simple tool to extract information about the name of households, the estimated radius (0.5 km, 1 km and 2 km) radius from the Lake Basin Mall. We held meetings with CHVs and took them through the descriptions of household characteristics as indicated in Table 1. The CHVs were thereafter provided with a form (attached with household identifiers) to classify all the households they provided into three categories; low (poor), middle and high (rich) based on the description in Table 1 and were asked to provide reasons for their choices. This list formed our sample frame. CHVs did not collect participant data on behalf of the study but only introduced our trained field workers to the households [26].

#### Field workers

A total of 36 field workers (FWs) were recruited based on at least one year of experience in conducting surveys, having a university degree in a related subject, and knowledge of the local language. Each FW was a resident of the same community in which they collected data. As such, the FWs were hypothesized to have prior knowledge and understanding of the living conditions of the study area (though, unlike CHVs, not of the specific households), but could also take a broader view as they had studied or lived outside their communities. The FWs used CommCare [30] to collect household data. During their visits, they classified household SES as low, middle or high based on their observations following examples provided by the research team (Table 1).

#### MCA model

The MCA model utilized the household survey variables associated with SES as recommended by the equity toolkit [19] and those included in the survey. These included working status, type of dwelling, housing rental status, having electricity, the main source of water, the main source of energy for cooking, ownership of refrigerator if the household has reared animals and ownership or availability of private care (Appendix 1). The selection of these variables included in the model follows recommendations of the equity tool and variables included in the survey. MCA is an econometric multivariable regression model used to assess household SES and has recently become popular compared to principal component analysis [31]. This is because it generates higher weights for the assets and households are then distinctly classified into wealth quintiles. It creates a household index from a linear combination of household assets, utilities and characteristics [17]. MCA is used to analyze a set of observations described by a set of nominal variables. Each nominal variable comprises several levels, and each of these levels is coded as a binary variable [32]. A set of variables including household possessions (private car, reared animals), utilities (cooking fuel, main source of drinking water), and house characteristics (type of dwelling, rental status, working status, presence of electricity) were included in the MCA model to create compositive households SES index. Higher values of the scores are interpreted as higher SES and low values are interpreted as low SES [32]. Using the MCA model, households were classified into three categories (tertiles/tercile): low, middle and high SES. MCA was used as the comparator for this analysis but was not conceptualized as a gold standard. The MCA model classifies observations equally (33.3%).

### Analysis

The analysis was conducted in Stata version 14. A concurrent comparison of all three methods (CHVs, FWs, and MCA) was conducted as described below.

#### Reliability analysis using percentage agreement and Kappa statistics

Percentage agreement is the percentage of judgments where the FW or CHVs have agreed with the MCA model based on several judgments made. The Kappa statistic has been used to evaluate the reliability of performance indicators, measuring the level of agreement and disagreements between a set of observers. It measures the agreement between the evaluations of two examiners when both are rating the same objects. It describes agreement achieved beyond chance as a proportion of that agreement that is possible beyond chance [33]. The value of the Kappa statistic ranges from -1 to + 1, with larger values indicating better reliability. A value of 1 indicates perfect agreement; a value of 0 indicates that agreement is no better than chance. Generally, a Kappa > 0.60 is considered satisfactory**.**

#### Effect of misclassification on the risk of self-reported hypertension and diabetes

We compared the association of the three methods of classifying household SES on self-reported hypertension and diabetes in household members using crude prevalence rates [34]. One individual living in the house was asked if they had ever been told by a doctor that they are hypertensive or diabetic. Prevalence rates were generated using a generalized linear model with a robust variance error estimator. This was achieved by the inclusion of a poison distribution and log link function to improve crude odds ratios and 95% confidence intervals. Prevalence rates provide a more accurate measure of risk than odds ratios when the prevalence of the outcome is > 10% for cross-sectional studies [35].

#### Operational definitions of terms

- Misclassification rate: Total number of households misclassified divided by the total number of households sampled
- Sensitivity: Proportion of households classified as low, middle or high by MCA model and also classified into the same SES groups by CHVs or FWs.
- Specificity: Proportion of households NOT-classified as low, middle or high by MCA model and also NOT-classified into the same SES groups by CHVs or FWs.
- Accuracy rate: Total number of households correctly classified divided by the total number of households sampled
- Predictive value: Measure of success incorrectly classifying the SES status of households.
- Positive predictive value (PPV):: Proportion of households classified into specific SES by CHVs or FWs amongst those correctly classified by the MCA model.
- Negative predictive value (NPV):: Proportion of households NOT-classified into specific SES by CHVs or FWs amongst those correctly NOT-classified into SES by the MCA model

## Results

The classification of household SES by CHVs is presented in Table 2. It shows the distribution by the quadrants, SES and distance from the Landmark.

**Table 2:** Sample distribution of households classified by Community Health Volunteers (CHVs)

	**Distance**												
	0.5 km				1 km				2 km				
**Quadrant**	Low	Mid	High	Total	Low	Mid	High	Total	Low	Mid	High	Total	Total
NE	0	0	0	0	2	17	6	25	9	15	1	25	50
NW	7	3	1	11	6	6	0	12	9	13	5	27	50
SE	0	0	0	0	12	5	1	18	12	19	2	32	50
SW	0	0	0	0	0	0	0	0	23	26	2	50	50
Total	7	3	1	11	22	31	7	60	50	69	10	128	**200**

*NE* North East, *NW* North West, *SE* South East and *SW* South West

Out of the 200 households, 79 (39.5%) were classified as low SES, 103 (51.5%) as middle SES and 18 (9.0) as high SES.

The classification of household SES by field workers is presented in Table 3 shown by SES, distance and quadrants.

**Table 3:** Sample distribution of households classified by Field Workers

Distance	0.5 km				1 km				2 km				
Quadrant	Low	Mid	High	Total	Low	Mid	High	Total	Low	Mid	High	Total	TOTAL
NE	0	0	0	0	4	18	3	25	16	9	0	25	**50**
NW	9	2	1	12	4	6	0	10	7	8	6	21	**43**
SE	0	0	0	0	11	4	1	16	14	10	1	25	**41**
SW	0	0	0	0	0	0	0	0	27	19	0	46	**46**
TOTAL	9	2	1	12	19	28	4	51	64	46	7	117	**180**

*NE* North East, *NW* North West, *SE* South East and *SW* South West

Out of the 180 households, 92 (51.1%) were classified as low SES, 76 (42.2%) as middle SES and 12 (6.7%) as high SES.

### A comparative analysis of sensitivity, specificity, predictive values of SES classification methods

Results in Table 4 showed that compared to the MCA model, FWs had a 21.7% ([95%CI = 14.4%-31.4%]) chance of successfully classifying households into low SES, 32.8% (95%CI = 23.2%-44.3%) chance of successfully classifying households into middle SES, and zero chance of classifying households into high SES. The sensitivity of FWs classification into low SES was 33.3%, 41.6% into middle SES and was not sensitive to high SES classification.

**Table 4:** A comparative analysis of sensitivity, specificity, predictive values of SES classification methods

(*n*=200)	**Sensitivity**	**Specificity**	**PPV**	**NPV**
	**% (95% CI)**	**% (95% CI)**	**% (95% CI)**	**% (95% CI)**
**FW SES**				
Low	33.3(22.4-46.30)	40.0(31.5-49.1)	21.7(14.4-31.4)	54.6(43.9-64.7)
Middle	41.6(29.7-54.6)	57.5(48.4-66.1)	32.8(23.2-44.3)	66.3(56.6-74.8)
High	0(0)	90.0(83.1-94.3)	0(0)	64.3(56.6-71.2)
**CHV SES**				
Low	30.0(19.6-42.8)	48.3(39.4-57.3)	22.5(14.5-33.1)	58.0(48.0-67.3)
Middle	50.0(37.4-62.5)	50.8(41.8-59.7)	33.7(24.5-44.2)	67.0(56.6-75.9)
High	1.6(0.2-11.2)	91.7(85.1-95.5)	9.1(1.1-46.7)	65.0(57.5-71.9)

*PPV* Positive Predictive Value, *NPV* Negative Predictive Value, *CI* Confidence Interval

Results further established that CHVs had a 22.5% (95%CI = 14.5%-33.1%) chance of successfully classifying households into low SES, 32.8% (95%CI = 23.2%-44.3%) chance of successfully classifying households into middle SES and zero chance of classifying households into high SES. The sensitivity of FWs classification into low SES was 30.0% (95%CI = 19.6%-42.8%), 50.0% (95%CI = 37.4%-62.5%) into middle SES and less likely to be sensitive to high SES classification at 1.6% (95%CI = 0.2%-11.2%), Table 4.

### Household SES misclassification and accuracy by FWs And CHVs compared to MCA model

FWs had 62.3% of poor households, 47.8% of middle households and 40% of high households misclassified when results were compared to the MCA model. The results were comparable with CHVs accuracy rates where CHVs misclassifications into low, middle and high SES were 57.7%, 49.5% and 38.4% respectively, Table 5.

**Table 5:** Household SES misclassification and accuracy by FWs And CHVs Compared to MCA model

	**Accuracy (%)**	**Misclassification (%)**
**Field Workers SES**		
Low	37.7	62.3
Middle	52.2	47.8
High	60.0	40.0
**CHVs SES**		
Low	42.3	57.7
Middle	50.5	49.5
High	61.6	38.4

### Comparison of Kappa reliability analysis between FW, CHV and MCA SES classification

Comparing Kappa coefficients in S1, the result indicates poor reliability of SES classification of households by FWs and CHVs compared with MCA econometric models. The level of agreement was not statistically significant in low and middle SES both in FWs and CHVs. Classification of the high SES by FWs and CHVs was statistically significant with poor characteristics of agreement based on interpretation of kappa statistics in S1 (*P* < 0.05 and kappa statistics < 0.20).

### Risk estimation of household SES on self-reported hypertension

The result shows the association of SES classification and self-reported hypertension. The prevalence of hypertension reduced with increasing MAC SES (16.2%, 15.5% to 14.9%) in contrast to increased hypertension with increase FW SES (13.6%, 16.2 and 23.6%) or CHV SES (15.9%, 14.1% and 25%). The results were however not statistically significant (S2).

### Risk estimation of household SES on self-reported diabetes

The result in the table shows the association of SES classification and self-reported diabetes. The prevalence of diabetes reduced with increasing MAC SES (3.0%;1.2% to 1.4%) in contrast to increased hypertension with increase FW SES (0.8%, 2.7% and 4.8%) or CHV SES (0.9%, 3.1%).The results were however not statistically significant (S3).

## Discussion

### Main findings

In the classification of households, CHVs and FWs classified the majority of the households as middle and low SES and very few households as high SES. Similarities between the two approaches to household SES classification are visible and reflect a pictorial view of most households in emerging economies. In most developing countries, households from poor and middle-class form the largest base of the pyramid SES [36]. According to Kenya Demographic and Health Survey (KDHS), amongst households in urban areas of Kenya, 49% are in the richest national quintile, compared to only 4.9% of those living in rural areas.

The sensitivity and the specificity of the household SES classification by FWs and CHVs were tested against the MCA model. The findings established that FWs were relatively more sensitive than CHVs in the classification of classifying households into lower SES while CHVs were found to be more likely to classify households into middle SES. These findings concur with a study conducted in Agincourt, South Africa which indicated that SES had been less polarized and thus converged towards the middle class [37]. In this study, both FWs and CHVs approaches depicted low sensitivity in the classification of high SES. Specificity was moderate in both methods during the classification of low and middle SES households, while both approaches emerged the best in high SES specificity. Both methods were found to have a low level of positive predictive values across all three categories of SES. The lowest percentage level of PPV on the two approaches concurs with the highest percentage of NPV on the same methods along with all three levels (low, middle, and high). In terms of accuracy rates, the study found that FWs and CHVs were moderately similar in the SES classification of the households. However, a high percentage of misclassification was established in the FWs and CHVs on one hand in comparison to the MCA model classification. Bias, overestimation, or underestimation of the asset-based MCA model was perceived to be attributed to the lower level of accuracy with the FW or CHV classification. CHVs may have made their classification based on personal judgment, thus compromising the accuracy of the outcome classification compared to the MCA model. Kappa analysis was conducted on the household survey data to establish the reliability of FWs and CHVs compared to the MCA model. The findings revealed that there exist low levels of agreement in FWs and CHVs in the classification of households in SES. Kappa statistics interpretation depicted poor reliability of CHVs and FWs in the SES classification of the households.

All three methods of SES classification had no significant effect on the self-reported prevalence of hypertension or diabetes. However, there was a pattern of relationship that indicated a potential association with the SES classification results showing a higher SES with high prevalence when comparing CHV and FWs SES. The results are contrary to the findings by Lubotsky who reported a positive association between health status and economic status of the households where children come from [38]. It is also inconsistent with a finding from a study by Were et al. who reported a statistically significant association between malaria prevalence and SES [39] but the study was using MCA classification to associate SES with malaria, not FW or CHV approaches.

The finding of this study showed that there were no superior methods between FW or CHV approaches in the classification of the household from SES but when compared to the MCA model there were disparities. The two approaches were not sensitive to classifying households. The findings are similar to the findings drawn from South Africa that showed that SES computation using an absolute index, principal components analysis and MCA were all consistent in assessing people's SES [37].

### Limitations

Despite the study being conducted successfully, some limitations were beyond the control of the study team. For instance, the MCA model is a mathematical equation and relies on the data collected from the field by the FWs. When the asset and household characteristics data have concerns of standard quality, the MCA model may also result in bias and poor-quality outcomes. Since the CHVs were community-based, they were also subjective and prone to a lot of bias leading to lower sensitivity in the SES classification of the households. Despite FWs having high education level further enhanced with five days of training, they had a higher probability of being misguided and making arbitrary decisions based on the assessments of the household asset ownership. This paper has not been able to make a direct comparison with other more recent validated asset-based classification tools such as Equity Tool (https://www.equitytool.org/kenya/), as not all of the same information was collected.

## Conclusions

The main objective of this study was to compare different methods of classification of households by asset-based indicators and estimate the effect of such classification on risk estimations. The study finding indicated that compared to MCA, CHVs and or FWs judgments have low sensitivity and low levels of success incorrectly classifying households into SES. The misclassification of households into SES was quite high amongst the CHVs and FWs compared to MCA. CHVs classification was more inclined towards low SES while FWs were inclined towards middle SES. Both have had a very low chance of sensitivity in identifying high SES. The level of accuracy was better in the middle SES classification than in other classes when using all three methods. Despite high rates of misclassifications and levels of agreement, there were no statistically significant differences in risk estimation by each of the three methods, but this requires further evaluation. Without any clear method of classifying households in SES before a survey is conducted, participatory methods relying on CHVs or FWs judgment may still be the most feasible approach. The best approach may be using a combination of approaches, use of validated tools and evaluating the agreement between them before applying any risk estimation.

### Supplementary Information

**Additional file 1:** **Table S1.** Comparison of Kappa reliability analysis between FW, CHV and MCA SES classification. **Table S2. **Risk estimation of household SES on self-reported hypertension. **Table S3.** Risk estimation of household SES on self-reported diabetes.

## Data Availability

The datasets used and/or analyzed during the current study are available from the corresponding author on reasonable request.

## Appendix 1

### Assets used in the MCA model

**Table:**

Working status	Employed full-time (30 + hours/week), Employed part-time, Looking after home or family,Unemployed or on sick leave, Retired, Studying,Voluntary worker, Other
Type of dwelling	Bungalow, Flat, Maisonette, Swahili, Shanty, Manyatta/traditional house, Other(list)
Housing rental status	Owns, Pays Rent/lease, No rent with the consent of the owner, No rent squatting
Having electricity	Yes, No
The main source of water	Piped water (into dwelling or plot), Public tab/standpipe, Tube well/Borehole with pump, Dug well, Water from spring, Rainwater collection, Vendors (e.g. tanker or cart), Surface water (e.g. river, stream, pond), Bottled water, Other
Main cooking source of energy	Firewood, Electricity, Liquefied petroleum gas (LPG), Biogas, Kerosene, Charcoal, Straw/shrubs/grass, Animal dung, Agricultural crop residue, Other
Reared animals for household	Yes, No
Ownership of refrigerator	Yes, No
availability of private car	Yes, No

## Abbreviations

MCA: Multiple correspondence analysis
CHV: Community health workers
FW: Field Workers
PPV: Positive predictive value
NPV: Negative predictive value
