# Supplementary material for: Comparison of household socioeconomic status classification methods and effects on risk estimation: lessons from a natural experimental study, Kisumu, Western Kenya
Source: Int J Equity Health. 2022 Apr 9;21:47. doi: 10.1186/s12939-022-01652-1 (PMC8994881; doi:10.1186/s12939-022-01652-1)
Supplement: Supplementary file 1 — Additional file 1: Table S1. Comparison of Kappa reliability analysis between FW, CHV and MCA SES classification. Table S2. Risk estimation of household SES on self-reported hypertension. Table S3. Risk estimation of household SES on self-reported diabetes. [file 12939_2022_1652_MOESM1_ESM.docx]

**Table S1: Comparison of Kappa reliability analysis between FW, CHV and MCA SES classification**

|  | **LOW SES** | | | **MIDDLE SES** | | | **HIGH SES** | | |
| --- | --- | --- | --- | --- | --- | --- | --- | --- | --- |
|  | Agreement (%) | Kappa | P-Value | Agreement (%) | Kappa | P-Value | Agreement (%) | Kappa | P-Value |
| **FWs SES** | 12.31 | -0.05 | 0.9983 | 21.15 | 0.026 | 0.0800 | 4.62 | 0.023 | 0.0008 |
| **CHVs SES** | 13.08 | -0.07 | 0.9998 | 18.85 | 0.028 | 0.0619 | 6.15 | 0.032 | 0.0001 |
|  | Interpretation of Kappa Statistic: <0.2 =poor, 0.21-0.40=Fair; 0.41-0.60=moderate;0.61-0.80=Substantial and >0.80 = excellent | | | | | | | | |

**Table S2.** **Risk estimation of household SES on self-reported hypertension**

|  |  |  |  | **Hypertension(N=260)** | |  | |  |
| --- | --- | --- | --- | --- | --- | --- | --- | --- |
| Method | SES | **n** | **N** | **%** | **Crude**  **Prevalence ratio** | **95% CI** | | **p-value** |
| MCA SES | Low | 16 | 99 | 16.2 | Ref |  | |  |
|  | Middle | 13 | 84 | 15.5 | 0.95 | 0.57-1.62 | | 0.871 |
|  | High | 11 | 74 | 14.9 | 0.92 | 0.55-1.54 | | 0.751 |
| FWs SES | Low | 17 | 125 | 13.6 | Ref |  | |  |
|  | Middle | 18 | 111 | 16.2 | 1.19 | 0.39-3.56 | | 0.753 |
|  | High | 5 | 21 | 23.8 | 1.75 | 0.52-5.85 | | 0.364 |
| CHVs SES | Low | 18 | 113 | 15.9 | Ref |  | |  |
|  | Middle | 18 | 128 | 14.1 | 0.88 | 0.33-2.32 | | 0.800 |
|  | High | 4 | 16 | 25.0 | 1.56 | 0.44-5.57 | | 0.486 |
| MCA= Multiple Correspondence Analysis FW= Field Worker CHV= Community Health Volunteer Ref: reference category | | | | | | | | |
|  | | | | | | |  |  |

**Table S3 Risk estimation of household SES on self-reported diabetes**

|  |  |  | **Diabetes(N=260)** | | |  |  |
| --- | --- | --- | --- | --- | --- | --- | --- |
| Method | SES | **n** | **N** | **%** | **Prevalence**  **Ratio** | **95% CI** | **p-value** |
| MCA SES |  |  |  |  |  |  |  |
|  | Low | 3 | 99 | 3.0 | Ref |  |  |
|  | Middle | 1 | 85 | 1.2 | 0.38 | 0.04-3.20 | 0.374 |
|  | High | 1 | 74 | 1.4 | 0.44 | 0.10-1.84 | 0.261 |
| FWs SES |  |  |  |  |  |  |  |
|  | Low | 1 | 126 | 0.8 | Ref |  |  |
|  | Middle | 3 | 111 | 2.7 | 3.47 | 0.17-72.31 | 0.422 |
|  | High | 1 | 21 | 4.8 | 6.25 | 0.36-107.0 | 0.206 |
| CHVs SES |  |  |  |  |  |  |  |
|  | Low | 1 | 114 | 0.9 | Ref |  |  |
|  | Middle | 4 | 128 | 3.1 | 3.64 | 0.26-50.58 | 0.335 |
|  | High | 0 | 16 | 0 | N/A |  |  |
